# Thrombotic microangiopathy (TMA) associated with pregnancy: role of the clinical laboratory in differential diagnosis

**DOI:** 10.1515/almed-2024-0053

**Published:** 2024-04-26

**Authors:** Patricia Ramos Mayordomo, Marta Capilla Díez, Danay Areli Ticona Espinoza, María Verónica Torres Jaramillo, Nathalie Martínez Tejeda, Thalia Gloria Ticona Espinoza, Cristina Colmenero Calleja, Virginia Fraile Gutiérrez

**Affiliations:** 16918 Servicio de Análisis Clínicos, Hospital Universitario Río Hortega, Valladolid, Castilla y León, Spain; 16918 Servicio de Nefrología, Hospital Universitario Río Hortega, Valladolid, Castilla y León, Spain; Servicio de Medicina Intensiva, Hospital Universitario Río Hortega, Valladolid, Castilla y León, Spain

**Keywords:** microangiopathic hemolytic anemia, eculizumab, thrombotic microangiopathy, preeclampsia, HELLP syndrome, atypical hemolytic uremic syndrome

## Abstract

**Objectives:**

Thrombotic microangiopathy (TMA) is characterized by thrombocytopenia, microangiopathic hemolytic anemia and target organ damage. Pregnancy is associated with several forms of TMA, including preeclampsia (PE), HELLP syndrome, thrombotic thrombocytopenic purpura (TTP) and hemolytic uremic syndrome (HUS). When HUS is secondary to a deregulation of the alternative complement pathway, it is known as atypical HUS (aHUS). Differential diagnosis is challenging, as these forms share clinical characteristics. However, early diagnosis is crucial for a specific treatment to be established and improve prognosis.

**Case presentation:**

We present the case of a 43 year-old primiparous woman admitted to hospital for an urgent C-section at 33 gestational weeks due to a diagnosis of severe preeclampsia and fetal distress. In the immediate postpartum, the patient developed acute liver failure and anuric renal failure in the context of the HELLP syndrome, anemia, thrombocytopenia, arterial hypertension (HTN) and neurological deficit. TMA study and differential diagnosis confirmed pregnancy-associated aHUS. Treatment with eculizumab was initiated, with good response and progressive improvement of clinical and analytical parameters.

**Conclusions:**

aHUS is a rare multifactorial disease that used to be associated with high mortality rates before the advent of eculizumab. Due to challenging diagnosis, the clinical laboratory plays a major role in the differential diagnosis and management of the disease.

## Introduction

Delayed diagnosis of thromboic microangiopathies (TMA) during pregnancy or postpartum may negatively affect the mother and the unborn child. There are different forms of pregnancy-associated TMAs, including preeclampsia (PE); HELLP syndrome; thrombotic thrombocytopenic purpura (TTP); hemolytic uremic syndrome (HUS); acute fatty liver of pregnancy; antiphospholipid syndrome and even severe postpartum hemorrhages. Since these diseases share symptoms and signs, appropriate differential diagnosis becomes crucial [1, 2].

PE is characterized by multisystemic, progressive, endothelial lesions preceding clinical manifestations. This condition complicates 3 % of gestations and is a major cause of maternal and perinatal morbimortality [3]. In contrast, the HELLP syndrome is an obstetric complication characterized by hemolysis, elevation of liver enzymes and thrombocytopenia [4, 5]. It is a variant or complication of severe PE. Some authors, however, consider it an unrelated syndrome, as 15–20 % of cases are not associated with proteinuria or HTN [4].

The hemolytic uremic syndrome (HUS) is defined by the triad of non-immune microangiopathic hemolytic anemia, thrombocytopenia and acute renal failure (AKI). Histological lesions are characterized by the development of systemic TMA, which primarily affects intra-renal vessels. In most cases, HUS is caused by enteric infection by *Escherichia coli* producing Shiga toxin (STEC), leading to typical HUS. In around 10 % of cases, HUS results from a deregulation of the alternative complement pathway, which causes endothelial damage and systemic TMA phenomena. This type of HUS is called atypical HUS (aHUS) and is considered a rare disease [1, 6].

Laboratory tests ensure correct differential diagnosis and help establish early diagnosis and treatment, thereby reducing morbimortality. It is worth noting that a multidisciplinary approach improves patient care.

## Case presentation

A 43 year-old primiparous patient with a single kidney for ureterovesical reflux in childhood, with previously normal kidney, diagnosed with early PE at 25 gestational weeks. Angiogenic markers were: soluble fms-like tyrosine kinase-1 (sFlt-1): 13,901 pg/mL (24–28 weeks: 618–3,205 pg/mL), placental growth factor (PlGF): 56.22 pg/mL (24–28 weeks: 130–1,108 pg/mL), sFlt-1/PlGF ratio: 247.26 (20–34 weeks: >85 raises suspicion of early PE).

The patient was admitted to another hospital at 33 gestational weeks with a diagnosis of intrauterine growth retardation and severe PE. Fetal distress and sustained bradycardia were established and an urgent C-section was performed. Previous creatinine: 1.09 mg/dL (0.51–0.95 mg/dL); alanine aminotransferase (ALT): 30 U/L (10–49 U/L); hemoglobin: 12.1 g/dL (12–15 g/dL); and platelets: 328 × 10^9^/L (140–450 × 10^9^/L).

Following surgery, the patient developed high blood pressure, kidney function deterioration, severe liver failure and abnormal hematological results. Clinical and analytical deterioration exacerbated, with creatinine: 1.79 mg/dL (0.51–0.95 mg/dL); ALT: 266 U/L (10–49 U/L); AST: 621 U/L (≤34 U/L); lactate dehydrogenase (LDH): 1,201 U/L (120–246 U/L); procalcitonin: 64.5 ng/mL (≤0.5 ng/mL); lactate: 4.4 mmol/L (0.5–1.6 mmol/L); hemoglobin: 10.1 g/dL (12–15 g/dL); platelets: 39 × 10^9^/L (140–450 × 10^9^/L); and D-dimer: 35,200 ng/mL (0–500 ng/mL). In the light of the severe acute liver failure in a context of HELLP syndrome, our hospital was contacted (unit of reference for liver transplantation) and the patient was transferred to the Intensive Care Unit (ICU).

On suspicion of pregnancy-associated TMA, differential diagnosis was performed for early diagnosis, as the clinical and analytical characteristics of the patient were associated with severe diseases with high mortality and morbidity rates (Table 1) [4, 7–9]. 

**Table 1:** Differential diagnosis of preeclampsia (PE), HELLP syndrome, acute fatty liver of pregnancy, thrombotic thrombocytopenic purpura (TTP) and hemolytic uremic syndrome (HUS).

Signs and symptoms/lab tests	Preeclampsia (PE)	HELLP syndrome	Acute fatty liver of pregnancy	Thrombotic hrombocytopenic purpura	Hemolytic uremic syndrome
Hypertension	100 %	85 %	50 %	20–70 %	80–90 %
Proteinuria	100 %	85 %	30–50 %	Associated with hematuria	80–90 %
Hemolytic anemia	No	Severe 50–100 %	Not frequent	Severe 100 %	Severe 100 %
Lactate dehydrogenase	Variable	>600	Variable	>1,000	>1,000
Plateletopenia	>15 × 10^9^/L	>20 × 10^9^/L	>50 × 10^9^/L	<20 × 10^9^/L	>20 × 10^9^/L
Transaminases	−	+ +	+ +	+/−	+/−
Renal insufficiency	+/−	20 %	90–100 %	30 %	100 %
Hypoglycemia	No	No	Present severe	No	No
Disseminated intravascular coagulation	No	Rare	Frequent	Rare	Rare
ADAMTS-13 <10 %	Absent	Absent	Absent	Present	Absent
sFlT-1/PlGF	>85 (early PE) >110 (late PE)	>85	<38	<38	<38

PE, preeclampsia; sFlT-1/PlGF, soluble fms-like tyrosine kinase-1/placental growth factor. Modified from: Arigita M et al. [4].

Immune-mediated intravascular hemolysis and TMA screening was initiated. Elevation of LDH: 4,625 U/L (100–190 U/L); added to undetectable haptoglobin: <6 mg/dL (36–195 mg/dL); elevation of total bilirubin: 5.91 mg/dL (0.2–1.1 mg/dL); and the presence of schistocytes: 2 % confirmed intravascular hemolysis *in vivo*. However, negative results of direct and indirect Coombs Test ruled out autoimmune hemolytic anemia.

PLASMIC Score was calculated. This diagnostic tool is used for differential diagnosis of TTP and other TMAs in hospitals where ADAMTS-13 determination in <24 h is not available and it is performed in an external laboratory. PLASMIC Score was 4 (low risk: consider alternative diagnoses) and ADAMTS-13 activity: 15 %, which rules out TTP, as diagnosis is established if <5–10 % [1, 10, 11]. STEC was negative, thereby excluding typical HUS.

**Figure 1:**
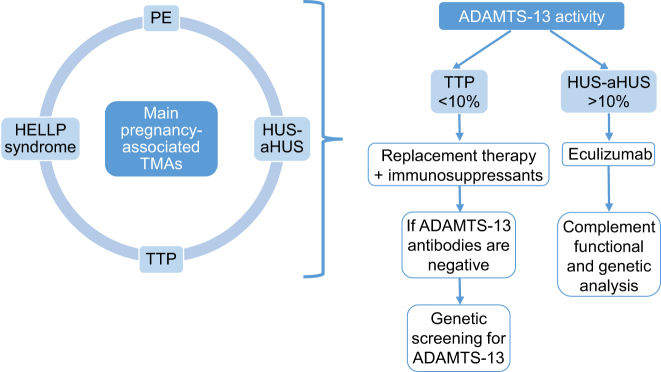
Summary of diagnostic criteria and treatment of choice according to ADAMTS-13 activity. PE, preeclampsia; TTP, thrombotic thrombocytopenic purpura; HUS, hemolytic uremic syndrome; aHUS, atypical hemolytic uremic syndrome.

The poor clinical course of the patient and need for life support since ICU admission (combined extracorporeal clearance and plasmapheresis), added to severe AKI with anuria, ADAMTS-13 >5–10 % and exclusion of other forms of TMA, led to diagnostic suspicion of pregnancy-associated aHUS [10]. Functional and genetic study of the complement were ordered to determine potential hyperactivity of the complement alternative pathway (considering component consumption or presence of activation markers) and treatment with eculizumab was initiated at day +5 (Figure 1) [1, 11, 12]. The clinical and analytical status of the patient improved. Kidney and liver function were restored, hemodynamics improved, hemolysis *in vivo* was discontinued, and extracorporeal clearance and plasmapheresis were no longer necessary since initiation of eculizumab therapy. It is worth noting that liver function parameters did not improve progressively, due to a complicated liver abscess, which was solved by drainage and targeted antibiotic therapy.

The analytical progress of the patient from admission to our hospital to discharge is shown in Table 2.

**Table 2:** Analytical evolution of the patient during her hospital stay in our hospital.

	Day												
Parameter	Post-C-section	+1	+2	+3	+4	+5	+10	+15	+ 20	+25	+30	+35	+40
Potasium, mmol/L (RV: 3.5–5.1)	5.8	3.6	4	3.4	2.9	3.4	3.8	4.6	3.6	3.1	3.4	3.1	3.8
Urea, mg/dL (RV: 12.8–42.8)	86.2	32.6	24	31.7	49.2	39.8	68.6	106.7	174.3	61	38.1	38	21.4
Creatinine, mg/dL (RV: 0.6–1.1)	3.17	1.62	1.18	1.6	2.25	1.67	0.95	1.07	2.05	0.83	0.73	0.46	0.43
AST, U/L (RV: 0–35)	5,473		2,136	614	265	165	83	131	182	111	85		
ALT, U/L (RV: 1–35)	3,925	1,344	1,509	590	281	149	53	108	104	46	28	25	
GGT, U/L (RV: 0–38)	114		116	116	112	78	90	468	852	957	796		
Total bilirubin, mg/dL (RV: 0.2–1.1)	5.91	6.87	8.45	9.74	12.45	14.53	18.28	14.06	18.28	16.15	13.13	13.04	
Direct bilirubin, mg/dL (RV: 0–0.2)	2.39	2.81	3.28	3.75	6.02	7.16	12.55	8	12.18	8.79	8.06	7.76	
Calculated indirect bilirubin, mg/dL (RV: 0–1)	3.52	4.06	5.17	5.99	6.43	7.37	5.73	6.06	6.1	7.36	5.07	5.28	
FA, U/L (RV: 30–120)	471		449	350	538	318	117	443	682	842	875		
LDH, U/L (RV: 100–190)	4,625	5,130		4,680	2,630	1,050	662	366		367	362		
Procalcitonin, ng/mL (RV: 0–0.5)	138.41		49.98	35.69	38.67	39.69	5.43	2.92	1.81	1.06	0.82	0.46	
Amonium, µmol/L (RV: 18–72)	184	111	290	79	105	78	100	41	70	56	44	8	
Hb, g/dL (RV: 11.4–15.1)	7.2	8.6	9.2	9	8.7	9.8	9.1	8.8	8.7	8.4	7.7	8	8
Free Hb, g/dL (RV: 0–0.1)		0.4		0.5	1.2	0.2	0.8	0.2	0.2	0.2	0.2		
Platelets, ×10^9^/L (RV: 150–350 × 10^9^/L)	27	50	63	69	47	10	25	67	216	186	167	260	374
D-dimer, ng/mL (RV: 0–500)	32,907			19,836									
Fibrinogen, mg/dL (RV: 180–420)	221	230	224	195	185	389	171	310	453	515	441	443	

RV, reference values; AST, aspartate aminotransferase; ALT, alanine aminotransferase; GGT, gamma-glutamyl transferase; ALP, alkaline phosphatase; LDH, lactate dehydrogenase; Hb: hemoglobin.

The results of the complement study were: C3: 34 mg/dL (70–150 mg/dL). C4: 2 mg/dL (14–60 mg/dL); CH50: <13 U/mL (42–95 U/mL); FH: 77 μg/mL (90–285 μg/mL); FI: 18 μg/mL (20–40 μg/mL); anti-FH antibodies: negative; and MCP: 90 % (77–121 %). C3 y C4, complement proteins synthesized in the liver, were significantly low which indicated liver failure. Therefore, there were no C3dg levels suggestive of C3 activation.

No complement regulatory gene mutations were identified in the 14-gene NGS panel (*CFH, CFHR1, CFHR2, CFHR3, CFHR4, CFHR5, C3, CFI, MCP (CD46), CFB, THBD, DGKE, CFP, ADAMTS13*), most associated with aHUS [13].

These results indicated severe PE and HELLP syndrome with hyperacute liver failure in the immediate postpartum. Suspicion of pregnancy-associated aHUS was raised based on the poor clinical course of the patient, persistence of hemolytic anemia, AKI with anuria, and exclusion of other conditions by differential diagnosis. However, predisposing gene mutations were not identified and, due to liver failure, levels of complement proteins synthesized in the liver decreased. Nonetheless, the clinical course and patient’s response to eculizumab therapy indicated pregnancy-associated aHUS. This clinical case suggests that there are triggers of complement hyperactivity, such as the activated proinflammatory and procoagulant phenotype of vascular endothelial cells, which shares clinical symptoms with those of primary aHUS induced by genetic abnormalities [14].

## Discussion

Diagnosis of TMA during pregnancy and postpartum is challenging due to the broad variability of the clinical manifestations of potentially overlapping entities. Since diagnosis is challenging, the clinical laboratory plays a major role in differential diagnosis and the management of the disease. Diagnosis of pregnancy-associated aHUS is based on a diagnosis of exclusion, after other causes of TMA are ruled out. Delayed diagnosis and failure to establish early treatment may be life-threatening.

aHUS is a rare, complex, multifactorial disease characterized by microangiopathic hemolytic anemia (hemoglobin <10 mg/dL; negative direct Coombs Test; elevated LDH; decreased haptoglobin; reticulocytosis; presence of schistocytes), thrombocytopenia (platelets <150 × 10^9^/L or decrease >25 % from baseline) and AKI [1, 2, 7, 8].

ALT, AST, LDH and potassium concentrations were the result of patient’s hemolysis *in vivo* rather than to hemolysis *in vitro* causing interference. Therefore, failure to report these results is considered malpractice. However, interference by this hemolysis causes a methodological interference that results in bilirubin underestimation.

**Figure 2:**
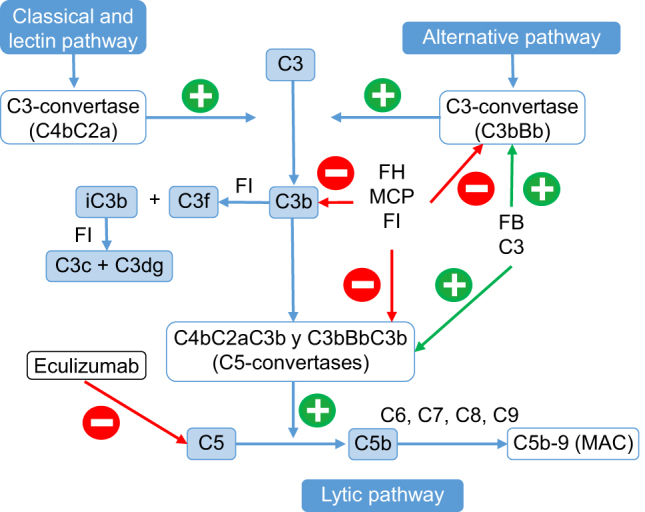
Complement system activation pathways and point of action of eculizumab in atypical uremic hemolytic syndrome (aHUS). Complement activation by the alternative, the classical or the lectin pathway causes the deposition of large amounts of C3b on the cellular membrane of the activator, hence leading to its opsonization and to C5 activation (lytic pathway), resulting in the formation of the membrane attack complex (MAC) and cellular lysis. MAC, membrane attack complex; FI, factor I; FH, factor H; FB, factor B; MCP, protein cofactor membrane.

The management of aHUS is based on the administration of eculizumab, a recombinant humanized monoclonal IgG2/4κ antibody that binds the protein complement C5, thereby inhibiting the cleavage of C5 and hence inhibiting the generation of the C5b-9 complex [15]. Deregulation of the complement alternative pathway leads to uncontrolled C5 inactivation, resulting in structural damage caused by the formation of the membrane attack complex. Blockade of the terminal pathway causes a rapid sustained reduction of this process (Figure 2) [1, 2, 12, 15].

Complement blockade with eculizumab is associated with a good clinical response and TMA reversal, significantly reducing inflammation, thrombotic risk, and levels of endothelial and organic damage biomarkers. This way, complement deregulation in the absence of genetic abnormalities probably plays a major role in the development of aHUS, as 40 % of patients do not have genetic sensitivity or anti-FH antibodies [1, 6, 11].

TMA classification is a matter of current research. As a result, constant advances in our understanding of the physiopathology of this disease keep TMA on the focus of the scientific community. Adopting a multidisciplinary approach in the management of rare diseases such as aHUS is crucial to establishing early diagnosis and improving prognosis.

## Lessons learned

– Diagnosis of pregnancy-associated TMA is challenging. The clinical laboratory plays a major role in differential diagnosis and management, due to the relevance of consultations to the laboratory from clinicians.
– Early management of aHUS with eculizumab has proven to improve prognosis.
– The risk for aHUS in subsequent pregnancy is around 25 % and decreases in the absence of complement gene abnormalities.
– The identification of hemolysis *in vivo* due to the clinical context and methodological analytical interference caused by this type hemolysis is essential; therefore, laboratory medicine specialists add value to the postanalytical phase by reporting laboratory results.
– A multidisciplinary approach provides clinical, social and economic benefits that significantly improve patient care standards.
